# Current Methods for Extraction and Concentration of Foodborne Bacteria with Glycan-Coated Magnetic Nanoparticles: A Review

**DOI:** 10.3390/bios12020112

**Published:** 2022-02-11

**Authors:** Emma Dester, Evangelyn Alocilja

**Affiliations:** 1 Nano-Biosensors Lab, Department of Biosystems and Agricultural Engineering, Michigan State University, East Lansing, MI 48824, USA; desterem@msu.edu; 2 Global Alliance for Rapid Diagnostics, Michigan State University, East Lansing, MI 48824, USA

**Keywords:** foodborne illness, glycans, glycoprotein, food safety, foodborne pathogens

## Abstract

Rapid and accurate food pathogen detection is an essential step to preventing foodborne illnesses. Before detection, removal of bacteria from the food matrix and concentration to detectable levels are often essential steps. Although many reviews discuss rapid concentration methods for foodborne pathogens, the use of glycan-coated magnetic nanoparticles (MNPs) is often omitted. This review seeks to analyze the potential of this technique as a rapid and cost-effective solution for concentration of bacteria directly from foods. The primary focus is the mechanism of glycan-coated MNP binding, as well as its current applications in concentration of foodborne pathogens. First, a background on the synthesis, properties, and applications of MNPs is provided. Second, synthesis of glycan-coated particles and their theorized mechanism for bacterial adhesion is described. Existing research into extraction of bacteria directly from food matrices is also analyzed. Finally, glycan-coated MNPs are compared to the magnetic separation technique of immunomagnetic separation (IMS) in terms of cost, time, and other factors. At its current state, glycan-coated MNPs require more research to fully identify the mechanism, potential for optimization, and extraction capabilities directly in food matrices. However, current research indicates glycan-coated MNPs are an incredibly cost-effective method for rapid food pathogen extraction and concentration.

## 1. Introduction

Foodborne disease outbreaks are a significant cause of illness and death, with over 3000 preventable deaths occurring annually in the United States alone [1]. These outbreaks can also strain the healthcare system, with over 48 million illnesses and 128,000 hospitalizations in the U.S. each year [1]. On a global scale, the World Health Organization estimates that foodborne pathogens are responsible for 600 million illnesses and 420,000 deaths annually, with the burden especially high for children under the age of 5 and individuals in low-income regions [2]. Approximately 20% of the U.S. population has increased vulnerability, including elderly, immunocompromised, or pregnant individuals [3,4]. These illnesses lead to severe economic losses, with one study estimating a cost of USD 7 billion to the U.S. economy as a result of food safety incidents [5].

Certain bacteria are commonly associated with foodborne outbreaks, including *Salmonella* spp., *Listeria monocytogenes*, *Bacillus cereus*, *Staphylococcus aureus*, and *Escherichia coli* [6]. These bacteria often have specific foods they thrive in, including fresh fruits and vegetables. The absence of a cooking step during fresh produce preparation, which can often kill bacteria, further increases risk for the consumer [7]. With these fresh foods highly recommended for a healthy diet, ensuring produce safety is of the utmost importance [8]. Products of animal origin, including meats, milk, and dairy products, are also commonly implicated in foodborne outbreaks [6].

Traditional foodborne pathogen detection methods are typically enumerative, meaning they are based on the growth of viable cells in nutrient-rich medium [9]. Detection can then proceed through continued growth of the bacteria in selective media to identify the presence of target pathogens. These preferred methods, outlined in the FDA’s Bacteriological Analytical Manual (BAM), are widely used but can take several days to produce conclusive results and incur high labor costs [10]. In recent years, rapid detection methods have been explored to remedy the time-consuming and costly nature of traditional enumerative methods. Nucleic acid-based quantitative analysis with real-time polymerase chain reaction (rt-PCR) can dramatically reduce detection time with high sensitivity [11]. However, high equipment costs are a significant limitation for some applications [12]. Biosensors, which convert biological, chemical, or biochemical elements into measurable signals, have also demonstrated success for rapid and cost-effective food pathogen detection [13].

Regardless of the detection method employed, concentration of bacteria from the food matrix is typically an essential step. The aforementioned conventional microbiological protocols often require overnight culturing of the bacteria (24–48 h) in selective media for pre-enrichment, increasing target bacterial concentration to detectable levels and minimizing interfering microorganisms [10]. For rapid extraction and detection methods, complex food matrices often introduce new challenges. Food macromolecules, including fats, glycogen, and other components, can block specific interactions between target molecules and receptors in many biosensors, as well as PCR-based detection [14,15]. Thus, there is an immense need for rapid and cost-effective techniques that concentrate target bacteria while effectively removing them from the interfering food matrix. Rapid detection methods such as biosensors and PCR often employ techniques such as centrifugation [16,17], filtration [17], dielectrophoresis [18], metal hydroxides [19,20], and magnetic nanoparticles (MNPs) [21,22,23,24] to attempt to fulfill these needs without overnight culturing (Table 1).

However, physical methods such as centrifugation and filtration often encounter challenges when separating bacteria from food matrices. Centrifugation, which separates particles based on density, can often fail to capture cells adhering to food particles while also capturing dead bacteria [25,26,27]. Filtration also encounters challenges in certain foods, leading to filter clogging and ineffective separation of bacteria [28,29]. Both methods lack specificity and cannot target specific bacteria species [26,30]. Similarly, metal hydroxides are non-specific and often have high limits of detection, or the assays require incubation before subsequent pathogen detection [19,20,30,31]. Some chemical methods allow for specificity and debris removal, such as dielectrophoresis [18,32,33,34]. However, the limit of detection for concentrated cells extracted from foods is often high, suggesting low capture efficiency through this method [18]. Thus, the search for a rapid, cost-effective, and efficient method of bacteria concentration from food matrices is still ongoing.

MNPs in particular have attracted attention for foodborne pathogen concentration due to their low cost, unique properties, and functionalization capabilities [35,36,37]. Antibody-functionalized MNPs, for example, have demonstrated specific and rapid concentration capabilities through immunomagnetic separation (IMS) [21,22]. Meanwhile, glycan-coated MNPs show promise as a lower-cost alternative for rapid food pathogen extraction and concentration through carbohydrate–lectin interactions [23,24]. In addition, application of glycan-coated MNPs in combination with biosensors is a cost-effective and rapid food pathogen detection method [38,39]. However, this methodology is often not included in conventional reviews of foodborne pathogen concentration methods [9,11,30,40]. This review seeks to summarize and analyze recent advancements and applications of glycan-coated MNPs for bacterial concentration from complex food matrices.

## 2. Magnetic Nanoparticles

Magnetic nanoparticles have attracted attention in recent years due to their unique properties. Nanoparticles, including MNPs, typically have a diameter ranging from 1–100 nm [44]. On this scale, particles can exhibit physicochemical properties disparate from those on a macro scale, including strength, magnetism, chemical reactivity, and optical properties, among many others [45]. In particular, the low cost and magnetic properties of MNPs have led to increased interest in their widespread use [35,36,37]. Due to these unique characteristics, MNPs have been applied to various roles in health, science, and technological innovation [46,47,48,49].

### 2.1. Synthesis and Characteristics

MNPs can be composed of a variety of materials including pure metals (Fe, Co, Ti, Ni), metal oxides, ferrites, and metal alloys. Iron oxides such as magnetite (Fe_3_O_4_) and maghemite (Fe_2_O_3_) are some of the most common MNP core materials [50,51]. Several synthesis methods for these nanoparticles include coprecipitation, high-temperature thermal decomposition, hydrothermal processes, and microemulsion, among many others [48,51]. An excellent review of these synthesis methods and materials, as well as their many biomedical applications, is detailed by Cardoso et al. [51].

One significant advantage of MNPs for many applications is their superparamagnetic properties, meaning they do not have a net magnetization and do not aggregate without an external magnet [24]. Superparamagnetism typically emerges at a particle size of 10–20 nm [52]. As a result, magnetic nanoparticles of this size quickly disperse in liquids but can still be magnetized and manipulated by an external magnetic field [24]. Thus, they are excellent tools for efficient capture of cells, proteins, and biomolecules. MNPs can easily be suspended in solutions for bacterial capture before separation from the supernatant using a magnet [53,54].

**Figure 1:**
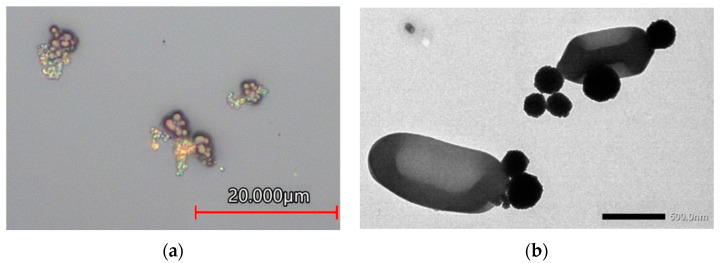
(**a**) Confocal laser microscope image of multiple MNPs bound to clusters of *S. aureus* cells; (**b**) TEM image of multiple MNPs bound to *L. monocytogenes* cells.

Another attractive aspect of MNPs is their high surface-area-to-volume ratio. The high adsorption capacity of this increased surface area leads to potentially high capture efficiency for the target cells [45]. In addition, MNPs are typically much smaller than bacterial cells, leading to multiple particles often attaching to a single cell. This behavior has been shown through microscopic imaging in multiple studies [24,42]. Similar TEM and confocal laser microscope images have been captured in the Nano-Biosensors Laboratory, pictured in Figure 1. This adhesion of multiple MNPs to a single cell may increase the probability of bacterial capture.

### 2.2. Surface Modification

Various natural polymer materials or alkene monomer copolymers are used to “coat” the nanoparticles for stabilization, modification, or the introduction of active groups [43]. Since uncoated metallic nanoparticles are chemically active and easily oxidized in air, protection and stabilization methods are often necessary to maintain their magnetic properties and prevent particle aggregation [54]. This is typically accomplished by a “core–shell” formation through physical adsorption or covalent bonding of desired compounds to MNPs [55]. While the magnetic properties of MNPs are determined by the metallic core, surface coatings can be used to control MNP selectivity and other properties specific to various applications. Some common coatings include surfactant molecules, ligands, silica, and colloidal gold, each with their own applications and unique characteristics [44,49,55]. A comprehensive review of MNP coating methodologies and their biomedical applications is provided by Pryazhnikov et al. [55].

### 2.3. Applications of MNPs in Food Pathogen Extraction

**Figure 2:**
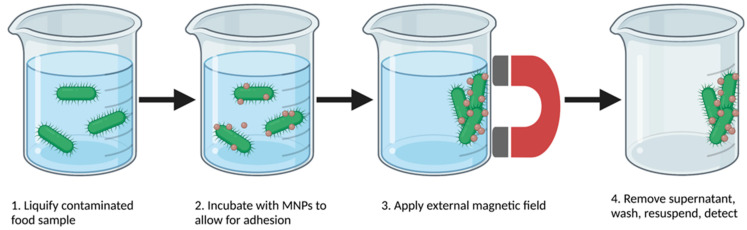
Overview of magnetic separation of bacteria from food samples.

In addition to the unique properties outlined previously, MNPs show immense promise in food pathogen detection due to their rapid and low-cost capabilities for the separation of bacteria from complex matrices without the need for centrifugation or filtration [45]. Although the functionalization and surface modification of MNPs for food pathogen extraction vary widely, most studies utilize the same basic procedure for bacterial extraction [24,39,56,57,58]. As shown in Figure 2, MNPs are introduced to a liquified food sample that is naturally or artificially contaminated with one or more bacterial species. After MNPs have been evenly dispersed in the liquid, the sample is incubated from one minute to up to an hour to allow the MNPs to adhere to the target cells. After incubation, an external magnet is applied, and the supernatant is removed. Samples can be resuspended in a lower sample volume for bacterial concentration, sometimes after multiple washing steps to remove any remaining food matrix components. Various detection methods using biosensors or PCR can then be implemented [39,56,57,58].

MNPs adhere to bacterial cells through a wide variety of mechanisms, often depending on their surface coating and functionalization. Common mechanisms include antigen-antibody binding [14,21,58,59], carbohydrate–lectin interactions [24,38,39,42,60], general electrostatic interactions [20,23,39], and covalent binding [61,62]. After concentration, these concentrated MNP/bacteria samples can be used in many detection methods, including PCR, cyclic voltammetry, enzyme-based assays, and other biosensors. The current state of standard magnetic separation techniques for food pathogen applications is outlined in the following sections.

## 3. Carbohydrate Functionalized (Glycan-Coated) Magnetic Separation

### 3.1. Mechanism of Glycan-Coated MNP Bacterial Adhesion

Glycans (complex carbohydrates) play an essential role in many cellular mechanisms, including cell–cell interactions [63]. Bacterial infection in particular is often initiated by interactions between bacterial surface proteins (e.g., lectins) and tissue carbohydrates [64], [41]. For instance, *H. pylori,* which causes chronic gastritis, often contains adhesins (lectins) on its cell surface that recognize host cell glycans to initiate infection [65]. Glycan–protein interactions can also play a role in the formation of bacterial biofilms through interactions between neighboring cells [66]. These interactions are non-covalent and electrostatic in nature, often consisting of van der Waals interactions as well as hydrogen bonds between hydroxyl and amino groups present on the carbohydrate and microbial protein surface [67,68].

In recent years, researchers have explored utilizing these protein–glycan interactions to extract and detect bacterial cells. Lectins have broad specificities for complex carbohydrates [68]. Thus, magnetic nanoparticles coated with glycans can bind to various bacteria cells through non-covalent electrostatic interactions with protein residues on the bacteria surface, effectively mimicking the role of cell surface glycans [53,63]. Due to the superparamagnetic properties of the MNPs, the MNP-bacteria complexes can then be manipulated by an external magnetic field, allowing for rapid extraction of the bacteria [23].

Nonspecific capture of bacterial cells may also be enhanced by the positively charged nature of some glycan-coated MNPs compared to the negatively charged bacteria cell membranes. The isoelectric points of both Gram-positive and Gram-negative bacteria range from 4.15 to 1.75, resulting in a net negative charge under physiological conditions [69,70]. This is due to negative cell wall components such as teichoic acids and lipopolysaccharides [71]. Thus, MNPs with net positively charged surface coatings may promote bacterial attraction and adhesion through electrostatic interactions with these negatively charged regions. Since glycans and proteins must be in close proximity to achieve adhesion, these general electrostatic interactions can promote bacterial capture by MNPs [72]. These electrostatic interactions are theorized to play a role in many glycan-coated MNP applications and are often improved through the addition of amino acids to the MNP coating [23,38,39].

**Figure 3:**
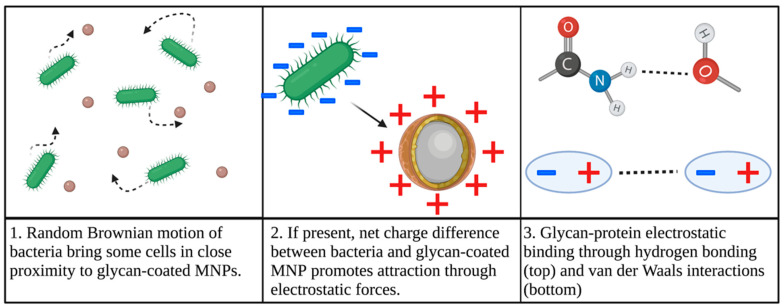
Overview of hypothesized mechanism for glycan-coated MNP binding to bacteria.

Thus, glycan-coated MNP binding to bacteria in a fluid matrix is hypothesized to be facilitated by a combination of forces, as summarized in Figure 3. Brownian motion, the random and uncontrolled movement of particles in a fluid, can initially facilitate movement that randomly brings cells in close proximity to glycan-coated MNPs [23,73]. If the coated nanoparticles have a net positive charge, electrostatic forces between MNPs and negatively charged cell membranes can also draw bacteria towards the MNPs [23,38,39,70]. Once in close proximity, due to random motion or electrostatic forces, the bacterium can adhere to the glycan surface of MNPs through non-covalent carbohydrate-protein binding, including van der Waals forces and hydrogen bonds [67,68]. A positively charged glycan coating may improve this adhesion by reducing electrostatic repulsion [23].

The general nature of these carbohydrate–protein interactions can best be illustrated through specific examples. Chitosan, for instance, is a cationic polysaccharide derived from chitin; due to its biodegradability and biocompatibility, along with other biological and physicochemical properties, it has become a favorable glycan coating for MNPs [36,74,75]. Chitosan has both hydroxyl (-OH) and amino (-NH_2_) groups. At a low pH, chitosan becomes positively charged through the protonation of these amino groups [37]. Thus, the chitosan-coated MNP will have a net positive charge at this pH, drawing the particles towards the negatively charged bacteria cells through generalized electrostatic interactions. Once the bacteria and MNPs are in close proximity, adhesion between chitosan and glycan-binding proteins on the bacterial surface can occur through non-covalent interactions. At a higher pH, however, binding may still occur. The negatively charged hydroxyl groups on chitosan can form hydrogen bonding interactions with positively charged pockets of the cell membrane, allowing for MNP-bacteria adhesion [24].

### 3.2. Glycan-Coated MNP Synthesis

As reviewed by Fratila et al. [76], carbohydrate coating of MNPs can be achieved through either an in situ process during MNP synthesis or as a post-synthetic functionalization step. For in situ methods, ligand adsorption onto the MNP surface is accomplished through MNP synthesis in the presence of carbohydrates. Post-synthetic methods entail the introduction of functionalized carbohydrates to the surface of the MNPs by ligand exchange, covalent linking, or non-covalent functionalization [76]. A wide variety of glycans have been employed as MNP coatings for attachment to bacteria. Some examples include mannose, galactose, fucose, and chitosan [23,42,65]. Glycan-coated MNPs may be further modified by adding other materials, such as amino acids, which could potentially be used to increase the positive charge of the MNP coating and promote bacterial adhesion [38,39].

### 3.3. Applications of Glycan-Coated MNPs

Extraction and concentration of bacteria using glycan-coated MNPs has been employed for a variety of applications outside of foodborne pathogens. Briceno et al. [23] utilized chitosan-coated iron oxide nanoparticles to concentrate *Mycobacterium* tuberculosis in sputum samples before detection with a colorimetric biosensing assay. The magnetically activated cell enrichment allowed for rapid detection of the bacteria at low concentrations in only 20 min. In addition to having 100% sensitivity and 99.7% specificity compared to the gold standard culture method, it was also a more cost-effective alternative at only USD 0.50/test [23]. Glycan-coated nanoparticles have also been employed to capture bacteria such as *Helicobacter pylori* [65] and *Pseudomonas aeruginosa* [77], among many others.

Glycan-coated MNPs have also been employed for extraction of pathogens directly from food matrices. One study utilized two unspecified forms of glycan-coated MNPs, referred to as F#1 and F#2, to extract *Salmonella Enteritidis, Escherichia coli O157:H7,* and *Bacillus cereus* from milk samples [24]. Magnetic nanoparticles were first added to 25 mL milk samples, followed by inoculation with concentrations of bacteria ranging from 2.9–4.5 log CFU/mL. After 10 min, the samples were mixed and magnetically separated before removal of the matrix and resuspension in 1 mL milk. Capture efficiencies ranged from 73–90% on a log basis, with no significant relationship between MNP type and milk or bacteria type. Notably, the researchers also tested the simultaneous extraction of all three bacteria. Capture efficiencies for each bacteria type in the mixture were similar to that of each bacteria species when extracted individually. Thus, this method was effective for the nonspecific extraction of multiple food pathogens [24].

The same author also successfully extracted *Salmonella Enteritidis, Escherichia coli O157:H7,* and *Listeria monocytogenes* from homogenized egg, vitamin D milk, and apple cider using cysteine-glycan coated iron oxide MNPs (F#2) [38]. In this study, the MNPs were affixed to plastic strips and inserted into 25 mL of the sample matrix instead of directly suspended in the matrix. The MNP strips were incubated in the matrix for 10 min before removal and subsequent pathogen detection using cyclic voltammetry. Although capture efficiency data was not available, this method distinguished between samples and negative controls with 95% confidence [38].

Another author created starch magnetic nanoparticles by co-crystallizing short chain glucans (SCG) with dextran-coated iron oxide nanoparticles [39]. The resulting glycan-MNPs were further modified through functionalization with the positively charged amino acid lysine. The authors used these strongly positive glycan-MNPs to electrostatically adhere to and extract *E. coli* O157:H7 cells from 10 mL samples of liquified sausage. The MNPs successfully adhered to and extracted 90% of the bacteria cells in only 10 min at concentrations from 10^1^ to 10^5^ CFU/mL in pure bacteria samples. In sausage samples, extraction and concentration combined with a colorimetric biosensor showed the 95% confidence limit of detection to be 30.8 CFU/mL. The nonspecific extraction method and specific biosensor detection method led to successful test results within food matrices [39].

Although glycan-coated MNPs can be used for nonspecific capture of bacteria, selectivity can also be improved by utilizing specific carbohydrate epitopes. For instance, biotinylated oligosaccharides immobilized to streptavidin-coated magnetic beads have been used to selectively capture *E. coli* strains with the *pap* pilus genotype [60]. After a 1-h incubation with the MNPs, bacteria samples suspended in phosphate-buffered saline solution (PBS) were magnetically separated for 5 min before resuspension. The assay had high selectivity for the three *pap-*containing uropathogenic (UPEC) strains when compared to three non-pathogenic or enterohemorrhagic *E. coli* strains. Capture efficiency was quantified through a BacTiter-Glo assay substrate. For the three target *E. coli* strains, capture efficiency ranged from 17–34% [60].

One primary advantage of carbohydrate functionalized MNPs is their low cost. One study, for instance, noted that using glycan-coated MNPs instead of a similar antibody-based assay reduced testing cost from USD 0.40 to USD 0.10 per assay [24]. In addition, glycan-coated MNPs do not require special handling and have a long shelf life at room temperature, further reducing overall expenses when compared to IMS [41]. The MNPs also typically require short incubation times (5–10 min) with the target bacteria [24,42]. Thus, the rapid and cost-effective nature of glycan-coated MNPs show promise for accessible extraction of foodborne bacteria without expensive equipment or long incubation times.

Despite its economical and efficient nature, this non-specific interaction may lead to complications in complex food matrices. Furthermore, due to their similar chemistries, carbohydrates present in the food may also electrostatically bind to the glycans on the MNPs [38]. Depending on the subsequent detection method employed, this may lead to limitations with pathogen detection. At its current state, the main drawback of this method for extraction of bacteria from food is the limited research (Table 2). Although other researchers have successfully extracted foodborne pathogens such as *E. coli* from pure cultures using glycan-coated MNPs [42,60], the previous papers by Matta and Alocilja [24] and You et al. [39] were the only research found referring to extraction of bacteria directly from a food matrix. Further study is required to determine the success of this method for food pathogen extraction.

## 4. Immunomagnetic Separation Versus Glycan-Coated MNP Separation

One of the most well-known and widely researched types of magnetic separation for foodborne pathogen extraction is immunomagnetic separation (IMS) [43]. Thus, a fuller understanding of glycan-coated MNP assays can be achieved by comparing and differentiating this method from IMS. Instead of glycans, IMS utilizes antibodies immobilized to MNPs to extract and concentrate target bacteria [43]. The differences between glycan-coated MNP extraction, however, are not limited to the surface coating. A summary of the key characteristics compared in this section are shown in Table 3.

### 4.1. Binding Mechanism

The first step of IMS is to capture the bacteria, which is facilitated by the binding of a specific antibody to the target bacteria. Antibodies are a three-lobed structure composed of two light chains and two heavy chains. Two of the lobes have fragment antigen binding (Fab) regions that are responsible for the specificity of the antibody [78]. These Fab regions adhere to antigens on the target cell surface through non-covalent electrostatic interactions of salt bridges and hydrogen bonds [79]. Long-range electrostatic forces can assist in bringing the antibodies towards the docking site [79].

Glycan-coated MNPs also adhere to bacteria through non-covalent electrostatic interactions, as discussed in detail in Section 3.1. In addition, the hypothesized long-range nonspecific electrostatic interactions are similar to those described for IMS. One important difference lies in the bond strength, with a single glycan–protein interaction having low affinity [72]. This can typically be overcome through the binding of multiple MNPs to a single bacterium [60]. Specificity of the two binding methods can also differ, as discussed in the following section.

### 4.2. Specificity

One of the most notable advantages of IMS is its high specificity; the use of antibodies allows for specific extraction of target bacteria and exclusion of natural microflora [21]. Specificity can be controlled through factors such as incubation time with the MNPs [80] and the specificity of the antibody itself [79]. The separation process can also remove PCR inhibitors, reducing purification steps required before detection [15].

Unlike IMS, current food pathogen extraction research using glycan-coated MNPs typically focuses on nonspecific concentration of cells [24,38,39]. However, some research has developed glycans specific to certain bacterial species [60]. Further, other studies have noted significantly different capture efficiencies between bacteria strains [42]. This versatility demonstrates the potential for glycan-coated MNPs to be applied in a variety of food pathogen detection settings, whether the target organism is known or unknown.

### 4.3. Experimental Time and Cost

IMS is rapid in comparison to many other assays, with incubation time (the time MNPs are exposed to the sample before magnetic separation) typically varying from 10–45 min [21,43,59,81,82]. Most sources successfully implemented the entire extraction procedure in under 2–3 h [14,58,59,81]. However, immunomagnetic separation is also costly compared to conventional methods [43]. Although this method could become more inexpensive through automation techniques that are currently being explored [14], the current lack of standardization does not permit its widespread implementation to detect foodborne pathogens at a low cost.

Glycan-coated MNP extraction is similar to IMS in its rapidity and may even shorten extraction times. Although limited data is available, currently published literature on foodborne pathogen extraction indicates only 5–10 min of incubation is necessary for glycan-bacteria binding to occur [38,39,42]. In addition, glycan-coated MNPs are significantly more cost-effective than immunomagnetic particles. As mentioned previously, one study conducted glycan-coated MNP extraction with 25% of the cost of a similar antibody-based assay [24].

### 4.4. Storage

After synthesis, the antibody-MNP complexes used in IMS must typically be stored at 4 °C until use [58,82,83]. Meanwhile, glycan-coated MNPs do not require special handling and have a long shelf life at room temperature [41]. For instance, one study examined the stability of iron oxide MNPs coated in either alginate or chitosan and suspended in buffer solutions [36]. The researchers found no flocculation, settling, or changes in hydrodynamic size after 6 months in room-temperature conditions. These simple storage measures further reduce overall expenses when compared to IMS. In addition, the simple storage conditions emphasize the feasibility of utilizing glycan-coated MNPs in low-resource settings, increasing the accessibility of rapid foodborne pathogen extraction.

### 4.5. Current Research

Immunomagnetic capture has been employed with a variety of bacteria and food matrices for detection using real-time or quantitative PCR. For instance, one author devised an IMS procedure for extracting strains of *L. monocytogenes* from inoculated soybean sprouts [59]. After a 20-min incubation with IM beads, the recovery was 1.16–1.96 logs below the initial concentration (approximately 1–10% recovery). With rt-PCR, this method was found to have a LOD of 4.4 log CFU/g [59]. Immunomagnetic separation of target bacteria from food has been employed with a variety of other detection methods with high sensitivity. One recent study, for example, was capable of detecting *E. coli* from fish muscle at concentrations as low as 10 CFU/mL using IMS and mass spectrometry [84]. Other detection methods implementing IMS for bacteria concentration include fluorescence methods [83], surface plasmon resonance [57,85], microfluidics [86], and other biosensors [22,87].

Standardization and automation of IMS has been tested. One study developed an automated IMS platform to extract and concentrate target bacteria using milk inoculated with *E. coli* O157:H7 [14]. After 60 min of sample pre-enrichment at 37 °C, immunomagnetic beads were added and incubated for 30 min. Results through plating of treated and untreated samples produced a capture efficiency of 20%; due to the initial 250 sample volume being reduced to 1 mL, their system led to a 100-fold concentration of the target bacteria. The entire extraction procedure could be completed in two hours [14]. This automated system was later tested alongside an enzyme-based colorimetric assay for bacteria detection [88]. With IMS, the assay could detect *E. coli* O157 in initial milk samples at concentrations as low as 3 × 10^2^ CFU/mL [88].

Although results are promising, current glycan-coated MNP research is more limited in comparison. However, the extraction method has been paired with cyclic voltammetry for successful pathogen detection [38]. Another study using poly-l-lysine-coated starch MNPs was able to detect *E. coli* O157 in sausage using a paper-based colorimetric biosensor with a limit of detection of 30.8 CFU/mL [39]. No current literature was found describing automation of this foodborne pathogen extraction method. However, due to their similar methodologies, an automated immunomagnetic separation system could easily be adapted for use with glycan-coated MNPs.

## 5. Discussion

At its current state, new methods for bacterial capture and concentration from foodborne pathogens have demonstrated several key advancements over the traditional culture method. New techniques with dielectrophoresis, centrifugation, filtration, and MNPs have been used to rapidly concentrate bacteria from foods to a detectable level without the need for cultural enrichment, often with assay times under 1 h in duration. In addition, the cost-effectiveness of many of these methods further enhances their applicability in food pathogen detection. However, physical methods such as centrifugation and filtration still show serious disadvantages in their lack of specificity and challenges with food matrix removal. Common chemical methods face challenges as well. Metal hydroxides lack specificity, and current methods in food matrices typically still require cultural enrichment before detection. Dielectrophoresis has demonstrated detection capabilities without cultural enrichment, but there is often a high limit of detection when only this concentration method is employed.

MNPs can provide cost-effective and rapid alternatives to many traditional food pathogen extraction methods while alleviating some of these concerns. Some applications, such as immunomagnetic separation, have been used in several assays in conjunction with PCR and biosensing techniques to rapidly detect bacterial concentrations in foods. Existing studies with glycan-coated MNPs have produced similar results, with the added advantage of reduced assay cost. In addition, the combination of lower-cost materials and room-temperature storage conditions enhances its accessibility and applicability.

However, the lack of standardization of MNPs hinders its current ability to be utilized as a food pathogen extraction method. Although immunomagnetic separation is the most researched technique, it still lacks standardization and automation that would allow for its widespread use. Meanwhile, there are few published works on direct bacteria extraction from food matrices using glycan-coated MNPs, and the composition of glycan coatings widely vary between studies. Regardless, MNPs show promise in a variety of fields in part due to their immense versatility and variability. The high capture efficiencies exhibited in multiple methods, as well as low limits of detection when combined with various biosensors and other detection methods, demonstrate their future potential.

## 6. Conclusions and Future Perspectives

Globally, foodborne pathogens are currently responsible for millions of illnesses and thousands of deaths each year. Thus, rapid and accurate detection methods are essential for reducing this strain on global health and the global economy. Although rapid detection methods are being developed to replace traditional culture-based methods, bacterial capture and concentration is still an important step for food pathogen detection. This review extensively outlined glycan-coated MNP methods for bacteria concentration in foods, which have shown promise for rapid detection of foodborne pathogens.

Although many reviews recount the applications of IMS, the application of other types of MNPs for foodborne pathogen concentration, such as glycan-coated MNPs, has not generally been discussed. Furthermore, reviews of glycan-coated MNPs rarely focus on their applications for foodborne pathogens. The qualities of these MNPs in particular, such as their low cost and room-temperature storage conditions, demonstrate the importance of including these techniques as a potential foodborne pathogen concentration method. Although current research in this application is limited, existing results are promising. Further, the mechanism of glycan-coated MNP binding to bacterial cells is rarely reviewed thoroughly in the literature. This review sought to compile sources on this binding mechanism to synthesize a comprehensive description of this mechanism and its specific applications in food. In future research, glycan-coated MNP applications in direct food pathogen bacteria extraction can be optimized, and bacterial adhesion mechanisms can be further elucidated. With these developments, MNPs have the potential to revolutionize current food pathogen detection methods to eliminate overnight incubation periods, reduce detection costs, and save lives.

## Figures and Tables

**Table 1:** Overview of existing methods for rapid bacteria concentration from food matrices.

Method	Description	Advantages	Disadvantages	References
Centrifugation	Bacteria concentrated by centrifugation and food solution can be removed	Can concentrate from large sample volume, 5–30 min assay	Not selective, loss of bacteria adhered to food particles, captures dead cells	[19,20,25,26,27]
Filtration	Samples passed through filters with various pore sizes, allowing bacteria to pass while eliminating food particles	1–10 min assay, remove inhibitors in food matrix	Filter clogging is common, non-target bacteria often concentrated	[17,28,29]
Metal hydroxides	Immobilization of titanium or zirconium hydroxides to bacteria through chelation followed by centrifugation	Cost-effective, maintains cell viability	Centrifuge required, needs enrichment step, limited research in foods	[19,20,30,31]
Dielectrophoresis	Nonuniform electric field used to manipulate bacterial cells	Option for specificity, maintains cell viability	Potentially low capture in foods	[18,32,33,34]
Glycan-coated MNP separation	Glycans on MNPs electrostatically bind, extract, and concentrate bacteria	Cost-effective, option for specificity	May bind to food particles, limited research in foods	[24,38,39,40,41,42]
Immunomagnetic separation	MNPs coated with specific antibodies bind, extract, and concentrate target bacteria	High specificity and capture efficiency	Costly synthesis and storage, not standardized	[14,21,22,43]

**Table 2:** Glycan-coated MNPs for extraction and concentration of foodborne pathogens.

Coating	Bacteria	Matrix	Capture	Detection Method	Source
Glycan (not specified), cysteine-glycan	*S. enteritidis*, *E. coli* O157:H7, *B. cereus*	Milk (vitamin D, reduced fat, fat-free)	73–90% *	N/A	[24]
Cysteine-glycan	*S. enteritidis, E. coli O157:H7, L. monocytogenes*	Homogenized egg, vitamin D milk, apple cider	N/A	Cyclic voltammetry	[38]
Lysine-SCGs	*E. coli* O157:H7	Sausage	>90% **	Colorimetric biosensor	[39]
Biotinylated oligosaccharides	*E. coli* (UPEC)	PBS	17–34%	N/A	[60]
Mannose Galactose	*E. coli* strains (3)	PBS	10–65% 15–75%	BacTiter-Glo assay	[42]

SCG: Short chain glucan, * log basis, ** Data for pure bacterial cultures, no capture efficiency data available for food samples.

**Table 3:** Immunomagnetic separation versus glycan-coated MNPs.

	Immunomagnetic Separation	Glycan-Coated MNPs
Binding mechanism	Antibodies on MNP surface bind to antigens on cell surface	Glycans on MNP surface bind to proteins on bacteria surface
Specificity	Very specific	Typically nonspecific, but specific glycans can be designed
Experimental Cost/Time	Rapid, relatively high cost	Rapid, low cost (25% of cost for IMS)
Storage	Antibodies require refrigeration	Room temperature
Current research	Well-researched and regularly used with PCR and biosensors	Limited studies in food matrices

## Data Availability

Not applicable.
